# Synergistic Killing of Polymyxin B in Combination With the Antineoplastic Drug Mitotane Against Polymyxin-Susceptible and -Resistant *Acinetobacter baumannii*: A Metabolomic Study

**DOI:** 10.3389/fphar.2018.00359

**Published:** 2018-04-16

**Authors:** Thien B. Tran, Phillip J. Bergen, Darren J. Creek, Tony Velkov, Jian Li

**Affiliations:** ^1^Monash Biomedicine Discovery Institute, Department of Microbiology, School of Biomedical Sciences, Faculty of Medicine, Nursing and Health Sciences, Monash University, Melbourne, VIC, Australia; ^2^Drug Delivery, Disposition and Dynamics, Monash Institute of Pharmaceutical Sciences, Monash University, Melbourne, VIC, Australia; ^3^Centre for Medicine Use and Safety, Monash Institute of Pharmaceutical Sciences, Monash University, Melbourne, VIC, Australia; ^4^Department of Pharmacology and Therapeutics, School of Biomedical Sciences, Faculty of Medicine, Dentistry and Health Sciences, The University of Melbourne, Parkville, VIC, Australia

**Keywords:** polymyxin, mitotane, combination therapy, multidrug-resistance, metabolomics

## Abstract

Polymyxins are currently used as the last-resort antibiotics against multidrug-resistant *Acinetobacter baumannii*. As resistance to polymyxins emerges in *A. baumannii* with monotherapy, combination therapy is often the only remaining treatment option. A novel approach is to employ the combination of polymyxin B with non-antibiotic drugs. In the present study, we employed metabolomics to investigate the synergistic mechanism of polymyxin B in combination with the antineoplastic drug mitotane against polymyxin-susceptible and -resistant *A. baumannii*. The metabolomes of four *A. baumannii* strains were analyzed following treatment with polymyxin B, mitotane and the combination. Polymyxin B monotherapy induced significant perturbation in glycerophospholipid (GPL) metabolism and histidine degradation pathways in polymyxin-susceptible strains, and minimal perturbation in polymyxin-resistant strains. Mitotane monotherapy induced minimal perturbation in the polymyxin-susceptible strains, but caused significant perturbation in GPL metabolism, pentose phosphate pathway and histidine degradation in the LPS-deficient polymyxin-resistant strain (FADDI-AB065). The polymyxin B – mitotane combination induced significant perturbation in all strains except the lipid A modified polymyxin-resistant FADDI-AB225 strain. For the polymyxin-susceptible strains, the combination therapy significantly perturbed GPL metabolism, pentose phosphate pathway, citric acid cycle, pyrimidine ribonucleotide biogenesis, guanine ribonucleotide biogenesis, and histidine degradation. Against FADDI-AB065, the combination significantly perturbed GPL metabolism, pentose phosphate pathway, citric acid cycle, and pyrimidine ribonucleotide biogenesis. Overall, these novel findings demonstrate that the disruption of the citric acid cycle and inhibition of nucleotide biogenesis are the key metabolic features associated with synergistic bacterial killing by the combination against polymyxin-susceptible and -resistant *A. baumannii*.

## Introduction

Multidrug-resistant (MDR) *Acinetobacter baumannii* has become a major global health threat (Talbot et al., 2006; Peleg et al., 2008; Boucher et al., 2009, 2013; Michalopoulos and Falagas, 2010). In 2013, the Centers for Disease Control and Prevention (CDC) classified *A. baumannii* as a “Serious threat” as approximately 63% of healthcare-associated *Acinetobacter* infections occurring in the United States were MDR (i.e., non-susceptible to ≥1 treating agent in ≥3 antimicrobial categories) (Magiorakos et al., 2012; Centers for Disease Control and Prevention [CDC], 2013). In 2017, carbapenem-resistant *A. baumannii* has been classified by the World Health Organization as one of the top priorities for research and development of new antibiotics, due to the current lack of novel antibiotic candidates in the drug development pipeline (Tacconelli and Magrini, 2017; WHO/EMP/IAU, 2017). Clearly, the development of novel therapeutic strategies to combat this deadly pathogen are urgently needed.

Polymyxin B and colistin are considered the last resort against MDR *A. baumannii* (Li et al., 2006a; Nation et al., 2015). Although polymyxins are believed to cause cell death primarily by disorganizing the Gram-negative outer membrane *via* binding to lipopolysaccharide (LPS), the precise antibacterial killing mechanism is not completely understood (Velkov et al., 2010; Trimble et al., 2016). Worryingly, we and others have demonstrated that polymyxin resistance rapidly emerges in polymyxin-susceptible *A. baumannii* following polymyxin monotherapy (Li et al., 2006b; Dudhani et al., 2010; Moffatt et al., 2010; Arroyo et al., 2011; Garonzik et al., 2011; Pelletier et al., 2013). *A. baumannii* becomes resistant to polymyxins by a reduction of the negative charge on the outer membrane (Velkov et al., 2010), which is achieved either through lipid A modification [with phosphoethanolamine (pEtN) and galactosamine (GalN) (Arroyo et al., 2005; Adams et al., 2009; Pelletier et al., 2013)] or loss of LPS (Moffatt et al., 2010).

Recently, the combination of an antibiotic (including polymyxins) and non-antibiotic drug has emerged as a potentially valuable and cost-effective approach to improve the clinical efficacy of currently available antibiotics against problematic MDR bacterial pathogens (Ejim et al., 2011; Hussein et al., 2016; Schneider et al., 2016, 2017). In a recent study, we demonstrated that the combination of clinically achievable concentrations of polymyxin B (2 mg/L) and the antineoplastic agent mitotane (4 mg/L) provided enhanced antimicrobial activity against MDR as well as polymyxin-resistant *A. baumannii* (Tran et al., 2018). Furthermore, the combination also prevented bacterial regrowth in polymyxin-susceptible strains. Mitotane is used for the treatment of adrenocortical carcinoma and Cushing’s syndrome (Lalli, 2015). Unlike other anticancer drugs that damage DNA and inhibit DNA replication, mitotane inhibits steroidogenesis in adrenocortical carcinoma cells (Lalli, 2015). The precise antimicrobial mechanism of mitotane, however, is presently unclear. Given the potential repositioning of mitotane to treat MDR *A. baumannii*, it is essential to understand the mechanisms by which the polymyxin/mitotane combination achieves this enhanced bacterial killing and suppression of bacterial regrowth.

Metabolomics has emerged as a valuable tool for elucidating the mechanisms of drug action in bacterial physiology and drug discovery (Mastrangelo et al., 2014; Vincent et al., 2016). Notably, metabolomics provides snapshots of cellular biochemical networks and helps explain how bacteria respond to antibiotic treatment at the systems level (Chen et al., 2007; Jia et al., 2009; Kaddurah-Daouk et al., 2014). Moreover, understanding how bacteria respond to antibiotic treatment at the metabolic level is valuable for the discovery of novel antibiotic targets (Jia et al., 2009). Accordingly, the primary aim of this study was to use untargeted metabolomics to elucidate the mechanism(s) of the enhanced antimicrobial activity of polymyxins against *A. baumannii* by mitotane.

## Materials and Methods

### Drugs and Bacterial Isolates

Polymyxin B (Beta Pharma, China, Batch number 20120204) solutions were prepared in Milli-Q^TM^ water (Millipore, Australia) and filtered through 0.22-μm syringe filters (Sartorius, Australia). Mitotane (Sigma-Aldrich, Australia, Lot number BCBG9480V) solutions were prepared in dimethyl sulfoxide (Sigma-Aldrich, Australia). All other reagents were purchased from Sigma-Aldrich (Australia) and were of the highest commercial grade available. Polymyxin-susceptible *A. baumannii* ATCC 17978 (polymyxin B MIC = 0.25 mg/L) and ATCC 19606 (polymyxin B MIC = 0.5 mg/L) were obtained from the American Type Culture Collection (Manassas, VA, United States). *A. baumannii* FADDI-AB225 (formally designated ATCC 17978-R2) is polymyxin-resistant (polymyxin B MIC = 16 mg/L) with phosphoethanolamine-modified lipid A and *pmrB* mutation derived from *A. baumannii* ATCC 17978 (Arroyo et al., 2011). *A. baumannii* FADDI-AB065 (formally designated ATCC 19606R) is a polymyxin-resistant (polymyxin B MIC = 64 mg/L), LPS-deficient, *lpxA* mutant derived from the ATCC 19606 strain (Moffatt et al., 2010). Isolates were stored in tryptone soy broth (Oxoid) with 20% glycerol (Ajax Finechem, Seven Hills, NSW, Australia) in cryovials at -80°C. Before use, *A. baumannii* ATCC 17978 and ATCC 19606 were subcultured onto nutrient agar plates (Media Preparation Unit, University of Melbourne, Melbourne, VIC, Australia) and *A. baumannii* FADDI-AB225 and FADDI-AB065 were subcultured onto Mueller–Hinton plates supplemented with 10 mg/L of polymyxin B (Media Preparation Unit) to maintain the selection pressure.

### Bacterial Culture Preparation for Metabolomics Experiments

To investigate the possible molecular mechanisms of polymyxin B and mitotane combination, we employed untargeted metabolomics to determine the changes in different metabolite levels in all *A. baumannii* strains following 2-h treatment with 2 mg/L polymyxin B, 4 mg/L mitotane, or the combination. To ensure the clinical relevance of these findings, the concentrations of polymyxin B (2 mg/L) and mitotane (4 mg/L) employed were within the clinically achievable range of concentrations of each agent (Hermsen et al., 2011; Sandri et al., 2013). A 2 h exposure to the antibiotics was selected for investigation, as extensive bacterial killing normally occurs with polymyxins across this time (Li et al., 2006b; Owen et al., 2007; Percin et al., 2014). For each *A. baumannii* strain, single colonies grown on nutrient or Mueller–Hinton agar were selected and grown overnight (16–18 h) in 20 mL cation-adjusted Mueller–Hinton broth (CAMHB; Oxoid, England; 20–25 mg/L Ca^2+^ and 10–12.5 mg/L Mg^2+^) in 50 mL Falcon tubes (Thermo Fisher, Australia) incubated in a shaking water bath at 37°C (shaking speed, 180 rpm). Following overnight incubation, each culture was transferred to a 1,000 mL conical flask with 250 mL of fresh CAMHB at ∼50- to 100-fold dilutions. Flasks were incubated at 37°C with shaking at 180 rpm for ∼3–4 h to log-phase (OD_600_ ∼0.5). Cultures (50 mL) were transferred to four 500 mL conical flasks and solutions of polymyxin B, mitotane, or both added to three of four flasks to give a final concentration of 2 mg/L for polymyxin B and 4 mg/L for mitotane; the remaining flask acted as a drug-free control. To prevent excessive bacterial killing, the starting bacterial inoculum used was ∼10^8^ cfu/mL. The flasks were further incubated at 37°C with shaking at 180 rpm. After 2 h, the OD_600_ reading for each flask was measured and normalized to ∼0.5 with fresh CAMHB and 10 mL samples transferred to 15 mL Falcon tubes (Thermo Fisher, Australia) for metabolite extraction. To minimize inherent random variation, for each strain four biological samples were prepared for each treatment condition.

### Metabolite Extraction for Metabolomic Studies

Following bacterial culture preparation, extraction of metabolites was immediately performed to minimize further drug effects on metabolite levels. Samples were initially centrifuged at 3,220 × *g* at 4°C for 10 min. Supernatants were then removed and bacterial pellets washed twice in 2 mL of cold normal saline followed by centrifugation at 3,220 × *g* at 4°C for 5 min to remove residual extracellular metabolites and medium components. The washed pellets were then resuspended with cold chloroform:methanol:water (CMW; 1:3:1, v/v) extraction solvent containing 1 μM each of the internal standards (CHAPS, CAPS, PIPES, and TRIS). The selected internal standards are physicochemically diverse small molecules not naturally occurring in any microorganism. Samples were then thrice frozen in liquid nitrogen, thawed on ice and vortexed to release the intracellular metabolites. After the third cycle samples were centrifuged for 10 min at 3,220 × *g* at 4°C, whereby 300 μL of the supernatants was transferred to 1.5 mL Eppendorf tubes for immediate storage at -80°C. Prior to analysis samples were thawed and centrifuged at 14,000 × *g* at 4°C for 10 min to remove the presence of any particles, and 200 μL transferred into the injection vial for LC-MS analysis (described below). An equal volume of each sample was combined and used as a pooled quality control sample (QC); namely, a sample that contains all the analytes that will be encountered during the analysis (Gika et al., 2007).

### LC-MS Analysis

Metabolites were detected with hydrophilic interaction liquid chromatography (HILIC) – high-resolution mass spectrometry (HRMS) using a Dionex high-performance liquid chromatography (HPLC) system (RSLCU3000, Thermo Fisher) with a ZIC-pHILIC column (5 μm, polymeric, 150 mm × 4.6 mm; SeQuant, Merck). The system was coupled to a Q-Exactive Orbitrap mass spectrometer (Thermo Fisher) operated in both positive and negative electro-spray ionization (ESI) mode at 35,000 resolution with a detection range of 85 to 1,275 *m/z*. The LC solvents were (A) 20 mM ammonium carbonate and (B) acetonitrile, operated via a multi-step gradient system. The gradient system at 80% B and was reduced to 50% B over 15 min, then reduced from 50% B to 5% B over 3 min, followed by wash with 5% B for another 3 min, and finally 8 min re-equilibration with 80% B at a flow rate of 0.3 mL/min (Zhang et al., 2012). The total run time was 32 min with an injection sample volume of 10 μL. All samples were analyzed as a single LC-MS batch to reduce the batch-to-batch variation. Mixtures of pure standards containing over 250 metabolites were also included in the analysis to aid metabolite identification.

### Data Processing, Bioinformatics, and Statistical Analyses

Conversion of LC-MS raw data to metabolites was conducted using IDEOM^1^ free software (Creek et al., 2012), which initially employed ProteoWizard to convert raw LC-MS data to mzXML format and XCMS to pick peaks to convert to peakML files (Smith et al., 2006; Scheltema et al., 2011). Mzmatch.R was subsequently used for the alignment of samples and the filtering of peaks using minimum detectable intensity of 100,000, relative standard deviation (RSD) of <0.5 (reproducibility), and peak shape (codadw) of >0.8. Mzmatch was also used to retrieve missing peaks and annotation of related peaks. Default IDEOM parameters were used to eliminate unwanted noise and artifact peaks. Loss or gain of a proton was corrected in negative and positive ESI mode, respectively, followed by putative identification of metabolites by the exact mass within 2 ppm. Retention times of authentic standards were used to confirm the identification of each metabolite (Level 1 identification based on MSI standards). Other metabolites were putatively identified (Level 2 identification based on MSI standards) using exact mass and predicted retention time based on the Kyoto Encyclopedia of Genes and Genomes (KEGG), MetaCyc, and LIPIDMAPS databases, using preference to bacterial metabolites annotated in EcoCyc. Raw peak intensity was used to quantify each metabolite. The free online tool MetaboAnalyst 3.0 was used for the statistical analysis. Briefly, putative metabolites with median RSD ≤ 0.2 (20%) within the QC group and IDEOM confidence level of ≥5 were incorporated into a table and uploaded to MetaboAnalyst. Data with >50% missing values were removed and remaining missing values replaced with half the minimum positive value in the original data. Data were filtered using interquartile range (IQR), normalized by the median, log_2_ transformed and auto scaled. Principal component analysis (PCA) was performed to identify and remove outliers. PCA is normally used to reduce the dimension of variables from a large data set (Ma and Dai, 2011). Outliers were defined as samples outside of ±2 standard deviations (SD) along the principal component 1 axis. One-way ANOVA was used to identify metabolites with significant level changes between all samples and Fisher’s least square difference (LSD) to determine the metabolites with significant level changes between treatment and control groups. Statistically significant metabolites were selected using a false discovery rate of ≤0.05 for one-way ANOVA and *p* ≤ 0.05 for Fisher’s LSD. KEGG mapper was used to determine the pathway modules by statistically significant metabolites containing the KEGG compound numbers.

## Results

### Multivariate and Univariate Analyses of the Metabolites Affected by Polymyxin B and Mitotane in *A. baumannii*

Untargeted metabolomics analysis using HILIC-based high resolution accurate mass LC-MS allowed detection of 1,769 putative metabolites in polymyxin-sensitive and -resistant strains of *A. baumannii* treated with polymyxin B and mitotane. The reproducibility of metabolite semi-quantitation was within acceptable limits based on the median RSD from independent biological replicates across the four *A. baumannii* strains, where the median RSD was 16% for all control groups and <20% for most treatment groups (**Table 1**) (Kirwan et al., 2014).

**Table 1:** Data precision of different treatment groups represented as the median relative standard deviation (RSD) for all assessed metabolites.

	Median RSD (%)
***A. baumannii* ATCC 17978**	
Control	16
Polymyxin B	17
Mitotane	21
Combination	16
***A. baumannii* FADDI-AB225**	
Control	16
Polymyxin B	18
Mitotane	16
Combination	23
***A. baumannii* ATCC 19606**	
Control	16
Polymyxin B	23
Mitotane	14
Combination	14
***A. baumannii* FADDI-AB065**	
Control	16
Polymyxin B	15
Mitotane	16
Combination	14
PBQCs	14

**FIGURE 1:**
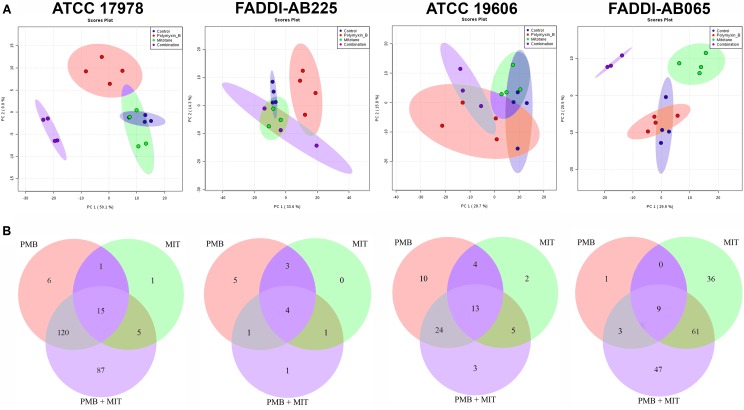
**(A)** PCA score plots showing metabolomic variance for untreated samples (blue) and samples treated with polymyxin B (red), mitotane (green) and combination (purple) for each *A. baumannii* strain along principal components 1 and 2. The degree of separation shows the level of variance between these groups. Principal component 1 axis is responsible for higher level of variance. **(B)** Venn diagrams showing the number of statistically significant metabolites affected by different treatments (one-way ANOVA, FDR ≤ 0.05; Fisher’s LSD, *p* ≤ 0.05) in each *A. baumannii* strain (PMB, polymyxin B; MIT, mitotane).

According to PCA, mitotane monotherapy clearly impacted the metabolome of FADDI-AB065 based on the first two principal components, but did not differentiate from controls for the other three tested strains (**Figure 1A**). Compared to polymyxin B and mitotane therapies, the combination produced more significant perturbations in the metabolomes of ATCC 17978 and FADDI-AB065 (**Figure 1A**). A minimal impact on the metabolome of FADDI-AB225 was observed for the combination (**Figure 1A**).

Polymyxin B monotherapy caused significant perturbations in a total of 142 metabolites in ATCC 17978, 51 in ATCC 19606, 13 in FADDI-AB225, and 13 in FADDI-AB065 (**Figure 1B**). For mitotane monotherapy, a total of 22 metabolites were perturbed in ATCC 17978, 24 in ATCC 19606, 8 in FADDI-AB225, and 106 in FADDI-AB065 (**Figure 1B**). The combination caused perturbations in a total of 227 metabolites in ATCC 17978, 45 in ATCC 19606, 7 in FADDI-AB225, and 120 in FADDI-AB065 (**Figure 1B**). Compared to mitotane monotherapy, polymyxin B monotherapy caused perturbation in more than twice the number of metabolites in the polymyxin-susceptible strains (ATCC 17978 and ATCC 19606). For the combination, over 50% of the perturbed metabolites in the polymyxin-susceptible strains were in common with those perturbed by polymyxin B and mitotane monotherapy. The common perturbed metabolites between combination therapy and polymyxin B monotherapy were much higher than the common perturbed metabolites between the combination therapy and mitotane monotherapy (**Figure 1B**). Although mitotane monotherapy had little impacts on the polymyxin-susceptible strains, it caused extensive metabolic changes in the LPS-deficient polymyxin-resistant FADDI-AB065 (**Figure 1B**). Most of the perturbed metabolites caused by the combination in this strain, consequently, were in common with those perturbed by mitotane monotherapy (**Figure 1B**).

**FIGURE 2:**
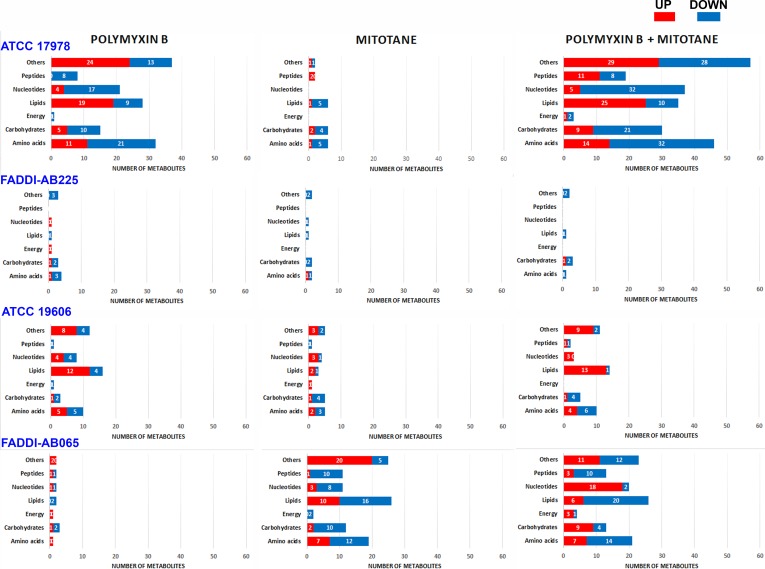
Bar graphs showing the number of significantly perturbed metabolites (ANOVA, FDR ≤ 0.05; Fisher’s LSD, *p* ≤ 0.05) in different metabolite classes following treatment with polymyxin B, mitotane, and the combination for polymyxin-susceptible *A. baumannii* ATCC 17978, polymyxin-resistant *A. baumannii* FADDI-AB225, polymyxin-susceptible *A. baumannii* ATCC 19606, and polymyxin-resistant *A. baumannii* FADDI-AB065. The class designated as ‘Others’ includes cofactors and vitamins, glycan, secondary metabolites and metabolites that could not be mapped into pathways based on existing bacterial metabolite databases.

The statistically significant metabolites impacted (one-way ANOVA, FDR ≤ 0.05; Fisher’s LSD, *p* ≤ 0.05) by different treatments in each *A. baumannii* strain were divided into seven different metabolite classes: amino acids, carbohydrates, energy, lipids, nucleotides, peptides, and others (the latter includes cofactors and vitamins, glycans, secondary metabolites and metabolites that could not be mapped into pathways). The number of metabolites impacted from each class that were higher or lower in abundance compared to the control group are shown in **Figure 2**. Details of all significantly impacted metabolites, including mass, retention time (RT), formula, putative identification, level of confidence (from IDEOM software), map, pathway, fold-change (FC; based on raw intensity), and FDR are shown in Supplementary Tables S1–S4 for all four strains.

### Significantly Impacted Lipids and Lipid Metabolites

**FIGURE 3:**
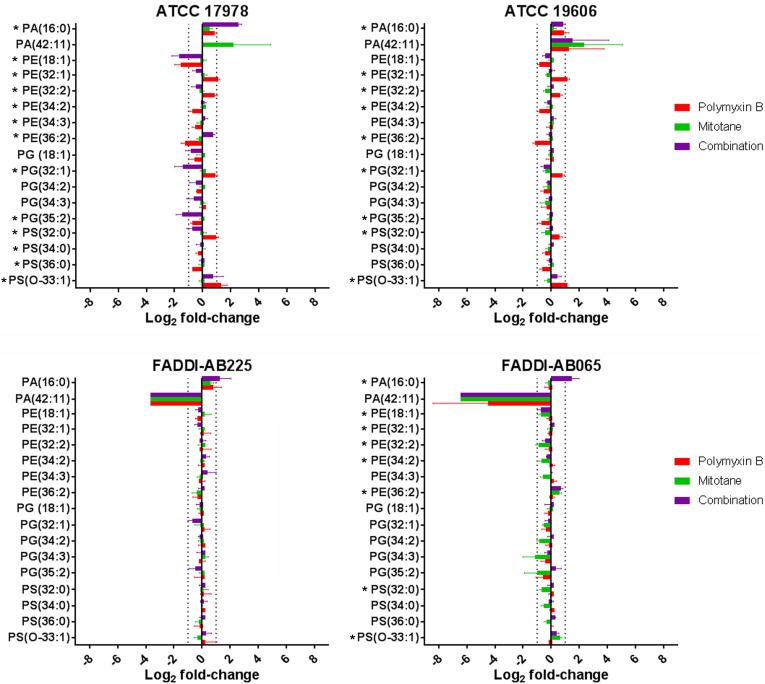
Glycerophospholipids in *A. baumannii* ATCC 17978, ATCC 19606, FADDI-AB065, and FADDI-AB225 following treatment with polymyxin B, mitotane and the combination. (^∗^one-way ANOVA, FDR ≤ 0.05; Fisher’s LSD, *p* ≤ 0.05, log_2_ fold-change ≥ |1| thresholds are indicated by vertical dotted lines).

Glycerophospholipids (GPLs) across four *A. baumannii* strains detected by LC-MS and their relative abundance (based on raw peak intensity) compared to the control groups are shown in **Figure 3**. In the polymyxin-susceptible strains, polymyxin B monotherapy induced significant changes in a wide range of GPL while mitotane monotherapy induced minimal changes. Overall, polymyxin B monotherapy caused a higher level of GPL perturbation in ATCC 17978 than ATCC 19606. Compared to polymyxin B monotherapy, the combination substantially enhanced the perturbation of putative lysophospholipids PA(16:0) and PG(35:2) in ATCC 17978 to greater than two-fold change in both cases. Against ATCC 19606, where the perturbation caused by polymyxin B monotherapy was lower than two-fold change in most cases, the combination did not significantly affect the GPL. In both polymyxin-resistant strains, polymyxin B monotherapy had a minimal impact on GLP, while mitotane monotherapy significantly affected a wide range of GPL in FADDI-AB065. In FADDI-AB065, mitotane monotherapy caused over two-fold reduction in putative GPLs PG(34:3) and PG(35:2). Interestingly, the combination did not enhance the lipid perturbation caused by mitotane monotherapy in FADDI-AB065.

**FIGURE 4:**
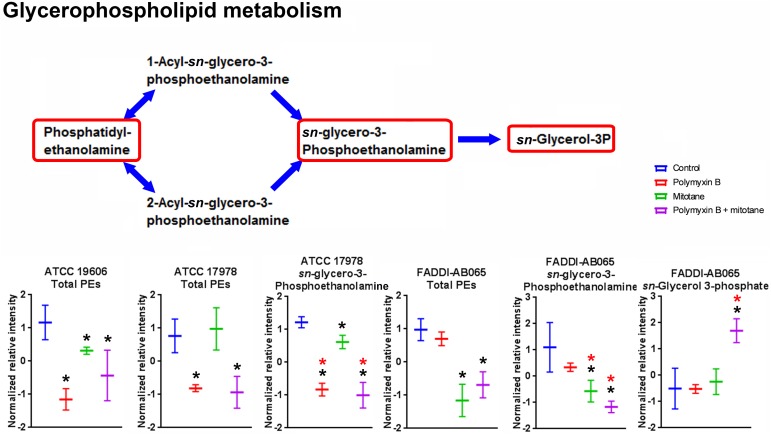
Metabolites involved in glycerophospholipid metabolism in *A. baumannii* significantly impacted by polymyxin B, mitotane, and the combination. Red boxes indicate statistically significant metabolites (Mean ± SD; ^∗^one-way ANOVA, FDR ≤ 0.05; Fisher’s LSD, *p* ≤ 0.05; ^∗^log_2_ fold-change ≥|1|).

The impact of polymyxin B and mitotane on the metabolites involved in GPL metabolism in *A. baumannii* is shown in **Figure 4**. In ATCC 19606, polymyxin B monotherapy and combination therapy caused statistically significant perturbation in total putative PEs (the sum of all detected putative PE species), although the changes in relative abundance were less than two-fold. Mitotane monotherapy had minimal impacts on these metabolites in ATCC 19606. For ATCC 17978, in addition to total putative PEs, polymyxin B monotherapy and combination therapy also substantially reduced putative *sn*-glycero-3-phosphoethanolamine (Log_2_FC = -2.42 and -2.83, respectively). Mitotane monotherapy only caused minor reduction of putative *sn*-glycero-3-phosphoethanolamine (Log_2_FC = -0.47) in ATCC 17978. Against polymyxin-resistant FADDI-AB065, polymyxin B monotherapy did not impact any metabolites involved in GPL metabolism. However, mitotane monotherapy caused significant perturbations in total putative PEs (Log_2_FC = -0.45) and putative *sn*-glycero-3-phosphoethanolamine (Log_2_FC = -1.71). The combination caused significant perturbations in total putative PEs (Log_2_FC = -0.34), putative *sn*-glycero-3-phosphoethanolamine (Log_2_FC = -1.62), and *sn*-glycerol-3-phosphate (Log_2_FC = 1.55).

**FIGURE 5:**
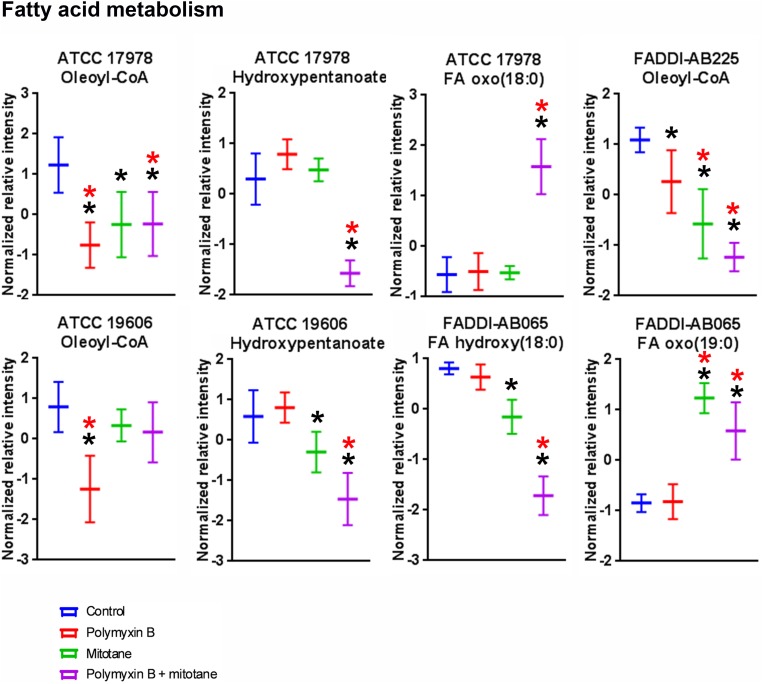
Putative fatty acyls from fatty acid metabolism in *A. baumannii* significantly impacted by polymyxin B, mitotane, and combination treatment (Mean ± SD; ^∗^one-way ANOVA, FDR ≤ 0.05; Fisher’s LSD, *p* ≤ 0.05; ^∗^log_2_ fold-change ≥ |1|).

The statistically significant (ANOVA, FDR ≤ 0.05, Fisher’s LSD, *p* ≤ 0.05) fatty acyls impacted by polymyxin B monotherapy, mitotane monotherapy, or the combination are shown in **Figure 5**. In ATCC 17978, ATCC 19606, and FADDI-AB225, polymyxin B monotherapy induced significant perturbations in putative oleoyl-CoA, a metabolite involved in fatty acid metabolism; notably, the relative abundance of oleoyl-CoA was over two-fold lower in polymyxin-susceptible strains treated with polymyxin B monotherapy compared to the untreated group. For mitotane monotherapy, significant reduction of oleoyl-CoA was observed for FADDI-AB225 and its parent strain ATCC 17978. Compared to polymyxin B monotherapy, the combination caused a greater reduction of oleoyl-CoA in polymyxin-resistant FADDI-AB225. In addition, the combination also caused significant perturbation of oxidized fatty acids including putative hydroxypentanoate in both polymyxin-susceptible strains, putative FA oxo(18:0) in ATCC 17978, and putative FA hydroxy(18:0) and putative FA oxo(19:0) in FADDI-AB065.

### Significantly Impacted Metabolites in Pentose Phosphate Pathway

**FIGURE 6:**
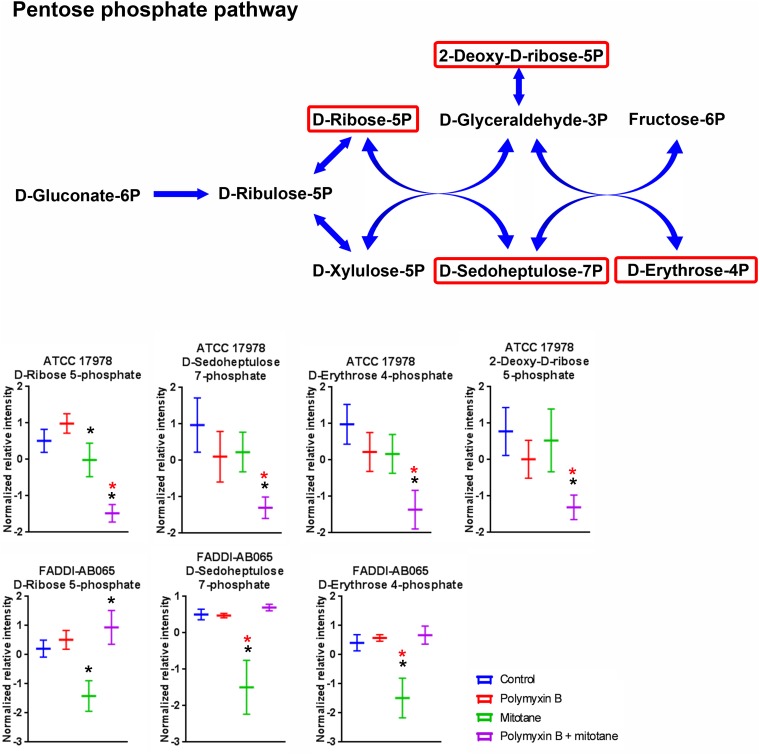
Metabolites in pentose phosphate pathway in *A. baumannii* significantly impacted by polymyxin B, mitotane, and the combination. Red boxes indicate statistically significant metabolites (Mean ± SD; ^∗^one-way ANOVA, FDR ≤ 0.05; Fisher’s LSD, *p* ≤ 0.05; ^∗^log_2_ fold-change ≥|1|).

Metabolites involved in pentose phosphate pathway of *A. baumannii* were perturbed by polymyxin B and mitotane (**Figure 6**). For ATCC 17978, polymyxin B monotherapy had no impact on metabolites of the pentose phosphate pathway; however, the combination therapy caused a significant reduction in D-ribose-5-phosphate, putative D-sedoheptulose-7-phosphate, D-erythrose-4-phosphate, and 2-deoxy-D-ribose-5-phosphate (Log_2_FC = -1.82, -3.09, -3.07, and -1.79, respectively). For FADDI-AB065, mitotane monotherapy caused a statistically significant reduction of D-ribose-5-phosphate, putative D-sedoheptulose-7-phosphate and D-erythrose-4-phosphate (≥2-fold change) (Log_2_FC = -0.94, -1.93, and -2.74, respectively).

### Significantly Impacted Metabolites in Citric Acid Cycle

**FIGURE 7:**
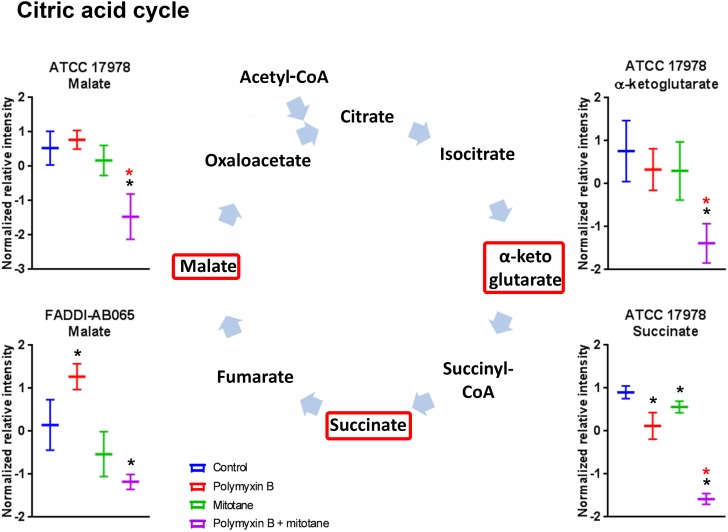
Metabolites in citric acid cycle in *A. baumannii* significantly impacted by polymyxin B, mitotane, and the combination. Red boxes indicate statistically significant metabolites (Mean ± SD; ^∗^one-way ANOVA, FDR ≤ 0.05; Fisher’s LSD, *p* ≤ 0.05; ^∗^log_2_ fold-change ≥|1|).

The impact of polymyxin B and mitotane on citric acid cycle in *A. baumannii* is shown in **Figure 7**. In ATCC 17978, succinate was significantly reduced by both polymyxin B and mitotane monotherapies; however, the highest level of reduction was observed with combination treatment (Log_2_FC = -2.49). In addition, the combination also caused a significant reduction in abundance in α-ketoglutarate and malate (Log_2_FC = -2.01 and -1.47, respectively) in ATCC 17978. The abundance of malate was also reduced by the combination treatment in FADDI-AB065 (Log_2_FC = -0.82), but increased by polymyxin B monotherapy (Log_2_FC = 0.82).

### Significantly Impacted Metabolites in Nucleotide Metabolism

**FIGURE 8:**
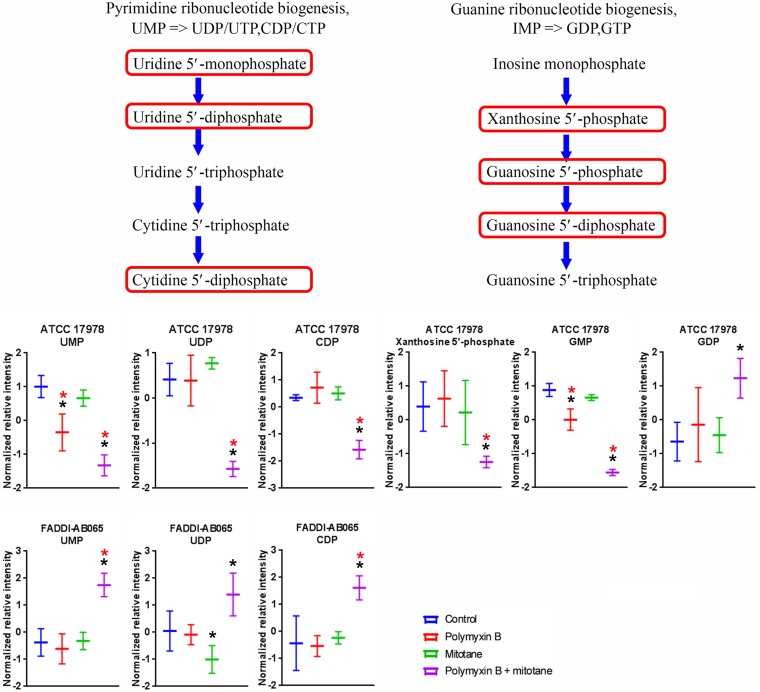
Metabolites in pyrimidine and guanine ribonucleotide biogenesis in *A. baumannii* significantly impacted by polymyxin B, mitotane, and the combination. Red boxes indicate statistically significant metabolites (Mean ± SD; ^∗^one-way ANOVA, FDR ≤ 0.05; Fisher’s LSD, *p* ≤ 0.05; ^∗^log_2_ fold-change ≥|1|).

A high number of metabolites involved in nucleotide metabolism in *A. baumannii* were significantly impacted by polymyxin B and mitotane alone and in combination (Supplementary Tables S1–S4). In both ATCC 17978 and FADDI-AB065, the pyrimidine ribonucleotide biogenesis pathway was over represented (≥2 metabolites in the module affected) (**Figure 8**). In ATCC 17978, UMP was significantly reduced by both polymyxin B monotherapy and the combination (Log_2_FC = -1.19 and -2.07, respectively); and the combination also reduced UDP and putative CDP (Log_2_FC = -1.47 and -1.57, respectively). In FADDI-AB065, UDP was slightly reduced by mitotane monotherapy, while increased by the combination therapy (Log_2_FC = -0.04 and 0.87, respectively). Only the combination caused increases in UMP and putative CDP (Log_2_FC = 1.74 and 1.33, respectively). A related pathway, guanine ribonucleotide biogenesis was also over represented in ATCC 17978. In this pathway, GMP abundance was significantly reduced by polymyxin B monotherapy and the combination (Log_2_FC = -1.28 and -3.22, respectively) in ATCC 17978, with greater perturbation caused by the combination. Additionally, only the combination impacted putative xanthosine 5′-phosphate (XMP) and GDP (Log_2_FC = -1.40 and -0.05, respectively), with greater perturbation occurring for putative XMP.

### Significantly Impacted Metabolites in Amino Acid Metabolism

**FIGURE 9:**
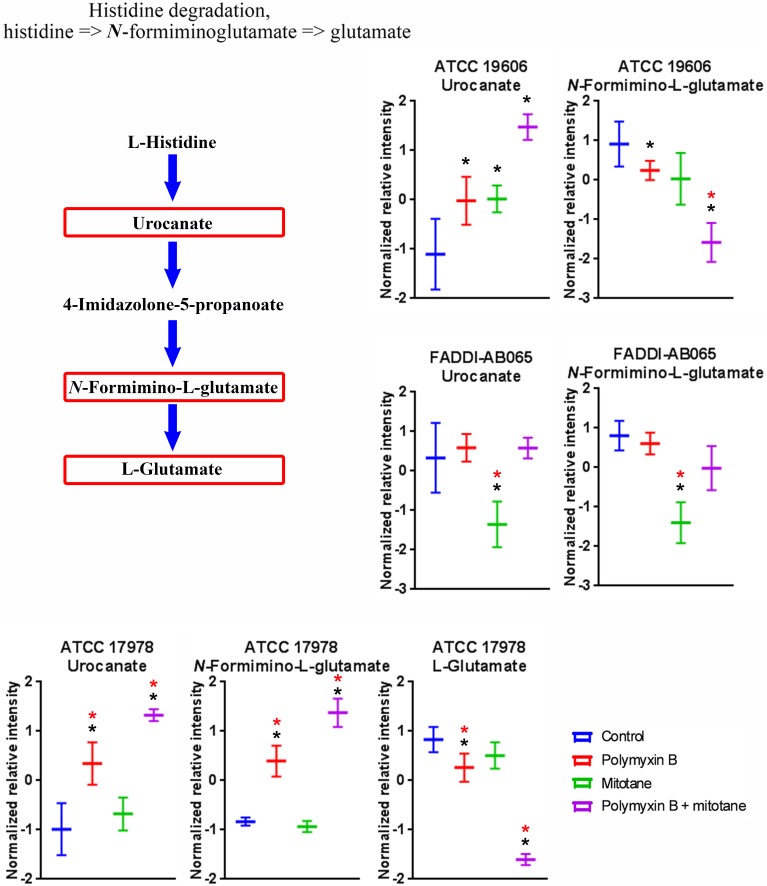
Metabolites in the histidine degradation pathway in *A. baumannii* significantly impacted by polymyxin B, mitotane, and the combination. Red boxes indicate statistically significant metabolites (Mean ± SD; ^∗^one-way ANOVA, FDR ≤ 0.05; Fisher’s LSD, *p* ≤ 0.05; ^∗^log_2_ fold-change ≥|1|).

Treatments with polymyxin B and mitotane alone and in combination caused significant perturbations to a high number of metabolites involved in amino acid metabolism in *A. baumannii* (Supplementary Tables S1–S4). Across ATCC 17978, ATCC 19606 and FADDI-AB065, histidine degradation was over-represented (≥2 metabolites in the module affected) (**Figure 9**). In ATCC 17978, polymyxin B monotherapy and the combination treatment caused significant perturbations in putative urocanate (Log_2_FC = 1.46 and 2.49, respectively), putative *N*-formimino-L-glutamate (Log_2_FC = 1.27 and 2.29, respectively) and L-glutamate (Log_2_FC = -1.25 and -4.44, respectively). The combination treatment, however, produced the highest level of perturbation in all three metabolites. In ATCC 19606, the intracellular concentration of putative urocanate was significantly increased by polymyxin B, mitotane, and combination treatment (Log_2_FC = 0.11, 0.30, and 0.71, respectively), with the highest level of perturbation observed with the combination. Putative *N*-formimino-L-glutamate was significantly reduced by combination therapy (Log_2_FC = -1.56). Interestingly, only mitotane monotherapy caused significant reduction in putative urocanate and putative *N*-formimino-L-glutamate in FADDI-AB065 (Log_2_FC = -1.13 and -1.75, respectively).

## Discussion

To improve the efficacy of polymyxins and prevent the emergence of resistance, novel polymyxin combination therapy against MDR *A. baumannii* is urgently needed (Pantopoulou et al., 2007; Pankuch et al., 2008, 2010; Lee et al., 2013; Ni et al., 2015). The combination of polymyxin B and the antineoplastic drug mitotane has been shown to produce synergistic bacterial killing and prevent regrowth in MDR *A. baumannii* (Tran et al., 2018). Mitotane is currently used for the treatment of adrenocortical carcinoma and Cushing’s syndrome where the mode of action is likely due to inhibition of sterol-*O*-acyl-transferase and inducing endoplasmic reticulum (ER) stress (Sbiera et al., 2015). However, the antibacterial mechanism of mitotane is unknown and has not been examined. Understanding the mechanism of the synergistic killing against *A. baumannii* by the combination of polymyxins and mitotane is essential for future repurposing of mitotane as an antimicrobial agent. This study is the first to investigate the potential antibacterial mechanism of this combination against *A. baumannii* using metabolomics. To ensure the observed responses were due to antibiotic treatments and not from extensive bacterial killing, a higher inoculum was employed for this metabolomics investigation.

It is well established that polymyxins exert their antimicrobial activity mainly through the disruption of the bacterial outer membrane (Hancock and Chapple, 1999; Hancock, 2001). Consequently, it was not unexpected that polymyxin B monotherapy impacted the membrane lipids of polymyxin-susceptible *A. baumannii* in the present study (**Figures 4**, **5**). Similar to a previous metabolomics study (Maifiah et al., 2017), pathway analysis revealed the majority of the significantly perturbed metabolites caused by polymyxin B monotherapy were in fatty acid and GPL metabolism (**Figures 4**, **5**). Our findings were also in agreement with a previous transcriptomic study that showed *A. baumannii* altered the expression of genes that are primarily associated with outer membrane biogenesis, fatty acid metabolism and phospholipid trafficking after 1-h exposure to colistin (Henry et al., 2015). Promisingly, the combination caused substantial reduction of *sn*-glycero-3-phosphoethanolamine in both polymyxin-susceptible and -resistant strains, while polymyxin B monotherapy only caused reduction of *sn*-glycero-3-phosphoethanolamine in the polymyxin-susceptible strain. This finding suggested that perturbation of *sn*-glycero-3-phosphoethanolamine in the polymyxin-resistant strains may be a synergistic killing mechanism of the combination.

In addition to the effect on the membrane lipids, pathway analysis also suggested that polymyxin B might affect the bacterial stress response through the degradation of L-histidine to L-glutamate. Since L-glutamate is an important metabolite involved in a wide range of bacterial metabolic processes including responses to acids and other stresses (Feehily and Karatzas, 2013), a reduced level of L-glutamate can stifle the stress response and result in cell death.

Remarkably, despite being a non-antibiotic, mitotane caused significant metabolic perturbation in the polymyxin-resistant strain lacking LPS (FADDI-AB065) (**Figures 1**, **2**). LPS is a key component of the outer membrane, a permeability barrier in Gram-negative bacteria (Nikaido, 2003); hence, the loss of LPS likely enables hydrophobic mitotane to cross the outer membrane and access its intracellular target(s). Surprisingly, pathway analysis showed that mitotane monotherapy also affected GPL metabolism. It is, however, unclear if this effect is connected to mitotane role in steroidogenesis inhibition in ACC cells. Mitotane also affected the histidine degradation pathway in the LPS-deficient strain. Unlike that observed with polymyxin B, the levels of both urocanate and *N*-formimino-L-glutamate of the histidine degradation pathway were significantly reduced by mitotane monotherapy (**Figure 9**). It is possible that mitotane upregulated the histidine degradation pathway to produce additional essential L-glutamate for stress response (Feehily and Karatzas, 2013).

Apart from its potential impact on membrane structure and the bacterial stress response, mitotane monotherapy also affected pentose phosphate pathway in FADDI-AB065 (**Figure 6**). Pentose phosphate pathway is responsible for the production of NADPH during the oxidative phase and ribose during the non-oxidative phase, which are essential products for anabolic reactions and DNA/RNA synthesis, respectively (Wamelink et al., 2008; Barcia-Vieitez and Ramos-Martinez, 2014). As the metabolites affected by mitotane in FADDI-AB065 are involved in the non-oxidative phase of pentose phosphate pathway, it is likely that mitotane also affects DNA/RNA synthesis in *A. baumannii*.

In combination, our findings showed polymyxin B and mitotane additionally affected citric acid cycle and nucleotide metabolism. Given the significant reduction of the majority of the metabolites involved in citric acid cycle, it is highly likely that the combination compromised energy production in *A. baumannii*. In relation to nucleotide metabolism, our findings were in line with the proposed effect of mitotane on RNA/DNA synthesis through perturbation of pentose phosphate pathway.

For antibiotic combination therapies, several models have been proposed to describe the mechanism of synergism, notably subpopulation synergy and mechanistic synergy (Bulitta et al., 2009; Landersdorfer et al., 2013). Subpopulation synergy refers to the killing of the resistant subpopulations of one drug by the second drug, while mechanistic synergy refers to the targeting of different cellular pathways of each drug (Landersdorfer et al., 2013). From the present study, it is likely that the synergism of polymyxin B and mitotane is a result of both mechanisms. Since FADDI-AB065 is a polymyxin-resistant derivative of ATCC 19606 following colistin monotherapy (Moffatt et al., 2010), FADDI-AB065 might represent a polymyxin-resistant subpopulation of ATCC 19606. Consequently, the susceptibility of FADDI-AB065 to mitotane monotherapy supports the subpopulation synergy model. Mechanistic synergy is suggested since much higher metabolic perturbation was observed with the combination therapy compared to polymyxin B and mitotane monotherapies. Additionally, given the lack of activity of mitotane alone compared to the enhanced activity in combination with polymyxin B, it is likely that bioavailability synergy, which refers to the increased intracellular availability of one drug due to the action of a second drug (Cokol et al., 2011), contributed to the enhanced activity observed.

The current findings suggest that non-antibiotic drugs that affect DNA synthesis, protein synthesis, and lipid synthesis may potentially be used in combination with a polymyxin in order to enhance bacterial killing.

## Conclusion

The present study is the first to investigate the synergistic killing mechanism of polymyxin B and mitotane in combination against *A. baumannii*. In addition to effects on lipid metabolism pathways identified in our previous metabolomic studies with colistin, the histidine degradation pathway has been shown to be impacted by polymyxin B monotherapy. As monotherapy, mitotane impacted lipid metabolism, histidine degradation and pentose phosphate pathway in a LPS-deficient polymyxin-resistant *A. baumannii* strain. Citric acid cycle and nucleotide metabolism were impacted by the combination in all strains. The novel finding from this study is that polymyxin B treatment *per se* causes significant perturbations in cellular lipids and amino acid metabolism, specifically histidine degradation, all of which were further enhanced by mitotane leading ultimately to the depletion of nucleotides. This study provides valuable mechanistic insights into the synergistic antibacterial killing of polymyxin and mitotane combinations against MDR *A. baumannii*, and is important for the potential repositioning of mitotane for an antimicrobial indication in combination with polymyxins.

## Data Availability

The raw data supporting the conclusion of this manuscript will be made available by the authors, without undue reservation, to any qualified researcher.

## Author Contributions

TT carried out the main experiments, data analysis, and wrote the manuscript draft. PB participated in experimental design. DC and TV participated in data analysis. JL designed the project and guided all experimental designs and data analysis. All authors participated in manuscript revision. All authors read and approved the final manuscript.

## Disclaimer

The content is solely the responsibility of the authors and does not necessarily represent the official views of the National Institute of Allergy and Infectious Diseases or the National Institutes of Health.

## Conflict of Interest Statement

The authors declare that the research was conducted in the absence of any commercial or financial relationships that could be construed as a potential conflict of interest.

## Footnotes

1 http://mzmatch.sourceforge.net/ideom.php
